# Valorization of Kraft Lignins from Different Poplar Genotypes as Vegetable Oil Structuring Agents via Electrospinning for Biolubricant Applications

**DOI:** 10.1021/acssuschemeng.4c05013

**Published:** 2024-07-29

**Authors:** José F. Rubio-Valle, Concepción Valencia, M. Carmen Sánchez-Carrillo, José E. Martín-Alfonso, José M. Franco

**Affiliations:** Pro2TecS − Chemical Product and Process Technology Research Center, Department of Chemical Engineering and Materials Science, Universidad de Huelva, ETSI, Campus de “El Carmen”, 21071 Huelva, Spain

**Keywords:** lignin, eco-friendly lubricant, nanofiber, rheology

## Abstract

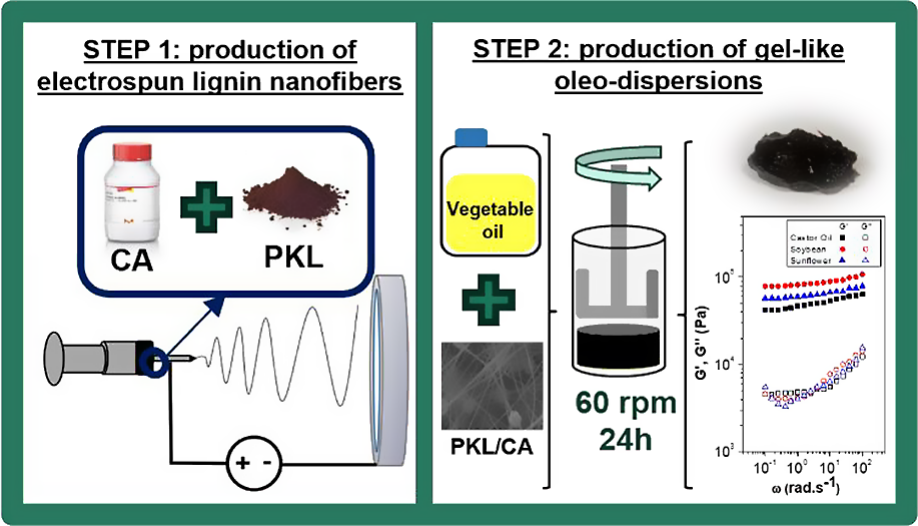

This work explores the use of Kraft lignins sourced from different poplar genotypes (*Populus alba* L. “PO-10-10-20” and *Populus* × *canadensis* “Ballotino”) isolated by selective acid precipitation (at pHs 5 and 2.5) to produce electrospun nanostructures that can be further employed for structuring vegetable oils. This approach offers a new avenue for converting these waste materials into high-value-added ingredients of eco-friendly structured lubricants. Electrospinning of poplar Kraft lignin (PKL)/cellulose acetate (CA) solutions yielded homogeneous beaded nanofiber mats that were able to generate stable dispersions when they were blended with different vegetable oils (castor, soybean, and high-oleic sunflower oils). Electrospun PKL/CA nanofiber mats with larger average fiber diameters were achieved using the lignins isolated at pH 5. Dispersions of PKL/CA nanofibers in vegetable oils presented gel-like viscoelastic characteristics and shear-thinning flow behavior, which slightly differ depending on the nanofiber morphological properties and can be tuned by selecting the poplar lignin genotype and precipitation pH. The rheological properties and tribological performance of PKL/CA nanofibers suitably dispersed in vegetable oils were found to be comparable to those obtained for conventional lubricating greases. Additionally, lignin nanofibers confer suitable oxidative stability to the ultimate formulations to different extents depending on the vegetable oil used.

## Abstract 2

Lignins from different poplar genotypes for the production of electrospun nanostructures and the subsequent promotion of the structuring of vegetable oils for lubricant applications.

## 1 Introduction

The manufacture of different multicomponent and fully formulated products may have a severe impact on the environment and global climate change.^1^ This impact depends largely on the type of raw materials and processing conditions.^2^ For this reason, nowadays there is a tendency to design new and innovative eco-friendly products based on renewable resources such as biopolymers as an alternative to fossil fuel-based polymers.^3,4^ In the lubricant industry, for instance, it is estimated that approximately 50–70% of the global lubricant production is discharged into the environment due to losses, spills, or accidents during the lifecycle stages, i.e. production, use and disposal as waste.^5,6^ Apart from replacing mineral or synthetic oils with vegetable oils or their derivatives in liquid lubricants, semisolid lubricants like greases typically contain relatively high contents (5–30 wt %) of oil thickeners, mainly metal soaps, and specifically lithium soaps (a key ingredient in roughly 85% of grease formulations).^7,8^ Although primary efforts to develop renewable grease formulations have been focused on replacing mineral oils with vegetable oils or glycerol esters while retaining the traditional metallic soap-based thickeners in the formulation,^9^ the substitution of these metallic soaps with renewable and/or biodegradable alternatives is also challenging within this industrial sector.^10−12^ This shift aims to ensure that greases maintain their functionality while mitigating their environmental impact.

Oil structuring with biopolymers has generated considerable interest, not only in the field of lubricants, but in a wide variety of applications.^13−17^ In previous studies, different strategies implying chemical modifications of biopolymers, as for instance by inserting isocyanate or epoxy moieties, were addressed to promote oil structuring via chemical cross-linking.^18,19^ Nevertheless, such chemical modifications often involve solvents and chemicals that make the production of biopolymers-derived oil structurants rather complex and not entirely eco-friendly. More recently, a simpler approach based on the use of electrospun biopolymer nanofibers has been implemented to physically structure oils.^20,21^ In these works, the morphology of the electrospun nanostructures were revealed to be the most influencing parameters for stabilizing the nanofiber dispersions and conferring appropriate gel-like characteristics, which basically occur through the formation of three-dimensional percolation networks.

On the other hand, within the biorefinery framework, which aims to produce chemical intermediates and a variety of end products from biomass, including consumer goods,^22^ the interest in lignocellulosic components as feedstocks in different chemical and energy industries has been progressively growing in the last decades. Moreover, in contrast to sugar- and starch-based biomass, lignocellulose is cheap, abundant, widespread, and not targeted for food consumption.^23^ A wide variety of forest species are inherently well-suited for producing lignocellulosic biomass. Among them, species of the Salicaceae family (poplars and willows) are naturally distributed throughout the northern hemisphere and have experienced significant development in genetic improvement that generates new highly productive hybrids.^24^

Lignin has been identified as a renewable resource with a high potential for industrial use.^25,26^ However, despite the fact that lignin output is currently estimated at around 40–50 t per year, its application to produce high added-value products is still very limited.^27,28^ On the contrary, it is considered a residue that, in most cases, is simply burned to obtain energy.^29^ In the pulp and paper industry, and particularly in the Kraft process, a lignin-rich but chemically heterogeneous fraction is obtained as a byproduct, which may require isolation or fractionation to some extent, especially when intended for high value-added applications.^29,30^ The isolation technique has an impact on the chemical composition and physical characteristics of lignin.^31,32^ Different strategies to perform the fractionation of lignin side streams include employing membrane technology for ultrafiltration,^33^ the targeted use of solvents,^34,35^ or the selective precipitation with acids.^36,37^ In the latter case, the pH of the black liquor is reduced by means of treatments with mineral acids thus causing the lignin to precipitate. In a previous study, Kraft lignins from different poplar genotypes were isolated by implementing a selective acid precipitation method (pHs 5 and 2.5) and further fully chemically characterized.^38^ It was shown that *Populus* × *canadensis* “Ballotino” genotype had a superior lignin content compared to the *Populus alba* L. “PO-10-10-20” genotype, because of differences in cell wall architecture and composition. In addition, the yield of lignin recovered varied as a function of precipitation pH, with the *Populus* × *canadensis* “Ballotino” genotype showing higher yields than *Populus alba* L., “PO-10-10-20” particularly at pH 2.5. These results suggested differences in susceptibility to delignification during Kraft pulping between the two genotypes. As a continuation of this research, we herein explore the potential of these lignins (obtained from different poplar genotypes and precipitated at different pH) to produce electrospun nanostructures, in combination with cellulose acetate as a cospinning polymer, and further promote the structuring of vegetable oils, thus providing a new pathway to valorize these waste materials. Moreover, this goal may represent an opportunity for certain industrial sectors, such as the lubricant sector, which is demanding the replacement of both traditional thickeners and petroleum-derived oils. With this aim, the resulting dispersions of lignin nanofibers in different vegetable oils were evaluated from rheological, tribological, and oxidative stability points of view.

## 2 Material and Methods

### 2.1 Materials

Four Kraft lignin samples (PKL) from two different poplar genotypes, namely *Populus alba* L. “PO-10-10-20” (PO) and *Populus* × *canadensis* “Ballotino” (Ba), isolated by selective precipitation (at pHs 5 and 2.5) were kindly provided by INIA-CSIC (Spain) and used as raw materials to produce electrospun nanofibers. Detailed information on the isolation procedure, composition, and chemical characteristics of these lignin samples can be found elsewhere.^38^ The most relevant compositional data and structural and chemical features of these samples are collected in Table 1. Cellulose acetate (CA) (39.8 wt % acetylated, *M*_n_, 30,000 g/mol), purchased from Merck Sigma-Aldrich S.A. (Germany), was employed as cospinning polymer. *N,N*-Dimethylformamide (DMF) and acetone (Ac) were also acquired from Merck Sigma-Aldrich S.A. (Germany) and used as solvents for electrospinning. Castor oil (CO) (dynamic viscosity: 550 mPa s at 25 °C and 26 mPa s at 90 °C) and soybean oil (SoyO) (dynamic viscosity: 55 mPa s at 25 °C and 8.6 mPa s at 90 °C) were purchased from Guinama (Spain). High-oleic sunflower oil (HOSO) (dynamic viscosity: 67 mPa s at 25 °C and 9.3 mPa s at 90 °C) was acquired from a local supermarket. The approximate fatty acid composition of these vegetable oils can be found elsewhere.^39^

**Table 1:** Total Lignin Content, Amount of Lignin β-O-4′ Substructures and Vinyl-Ether Expressed per 100 Aromatic Units (Expressed as Percentage of the Total Linkages), Weight-Average (*M*_w_) and Number-Average (*M*_n_) Molecular Weights, Polydispersity (*M*_w_/*M*_n_) and Total Phenol Content of Lignin Samples Studied (Data Taken from Ref (38))^a^

	PO-2.5	PO-5	Ba-2.5	Ba-5
total lignin content (%)	91.0	96.2	95.6	98.0
β-O-4′ substructures (%)	1.6	2.4	2.3	3.3
vinyl-ether (%)	2.3	2.5	0.3	0.6
*M*_w_ (Da)	5375	5595	5140	5305
*M*_n_ (Da)	4590	4485	4205	4180
*M*_w_/*M*_n_	1.21	1.19	1.22	1.27
total phenol content (mg GAE/g lignin)	588.7	644.5	529.1	638.9

a Codes applied to refer to these lignin samples are PO-2.5 and PO-5 for lignins isolated from the PO genotype at pHs 2.5 and 5, respectively, and Ba-2.5 and Ba-5 for lignins isolated from the Ba genotype at pHs 2.5 and 5, respectively.

### 2.2 Preparation of Electrospun PKL/CA Nanofiber Mats

PKL and CA were solubilized in a 1:2 v/v DMF/Ac blend, under magnetic stirring (500 rpm) for 24 h, at room temperature (22 ± 1 °C) fixing the total PKL/AC concentration (30 wt %) and PKL:CA weight ratio (70:30).

The PKL/CA solutions were electrospun in a chamber (Make: DOXA Microfluidics, Spain). These solutions were continuously fed into the electrospinning chamber at a controlled flow rate (0.6 mL/h), fitted with a plastic syringe containing a 21-G needle, and horizontally arranged, which was connected to a high-voltage power supply providing 17 kV. The electrospun nanofiber mats were collected on an aluminum plate at a distance of 15 cm from the needle tip. Electrospinning was carried out at room temperature and controlled relative humidity (45 ± 1%).

### 2.3 Dispersion of Nanofibers Mats in Vegetable Oils

Electrospun PKL/CA mats were carefully removed from the collector and subsequently dispersed in the vegetable oils, at a 15 wt % concentration. This concentration was chosen based on preliminary studies^40^ to provide gel-like rheological properties comparable to those of commercial lubricating greases. Suitable dispersions were easily achieved by applying a gentle mechanical agitation (60 rpm) for 24 h, at room temperature. Afterward, samples were stored at room temperature for further characterization.

### 2.4 Characterization Techniques

Electrospun nanofiber mats were examined in a JXA-8200 SuperProbe (Make: JEOL, Japan) scanning electron microscope (SEM) using a 15 kV acceleration voltage and ×1000 and ×4000 magnifications. The specimens were previously covered with gold in a BTT150 sputter coater (Make: HHV Ltd., UK).

The microstructure of the gel-like oleo-dispersions was also analyzed by SEM in an AURIGA (Make: Zeiss, USA) apparatus with a secondary electron detector at 20 kV acceleration voltage. The oleo-dispersions were previously subjected to a chemical fixation treatment^41^ and, subsequently, sputtered with a thin layer of gold.^42^ The FIJI ImageJ software was utilized to analyze SEM images and calculate the average fiber diameter. 100 random observations were conducted for each sample under the same magnification.^43^

The rheological properties of oleo-dispersions were investigated in a Rheoscope (Make: Thermo Scientific, USA) rheometer, using a serrated plate–plate measuring geometry (20 mm diameter, 1 mm gap). Small-amplitude oscillatory shear (SAOS) measurements were carried out in a 0.03–100 rad s^–1^ frequency range. The viscoelastic functions were monitored inside the linear viscoelastic region, which was previously determined by conducting stress sweep tests. In addition, viscous flow measurements were performed in the shear rate range of 10^–2^–10^2^ s^–1^. In general, all rheological measurements were done at 25 °C, however, some SAOS tests were occasionally carried out at 90 °C.

A Physica MCR-501 rheometer (Make: Anton Paar, Austria) fitted with a tribological cell was utilized to carry out the tribological characterization. The tribological cell comprised a 1/2″ steel ball that rotates on three rectangular steel plates inclined 45°, where electrospun lignin oleo-dispersions acting as lubricants were spread. The tribological study involved the determination of the friction coefficient as a function of the ball rotational speed, in a 0.1 to 1000 rpm range, fixing the temperature (25 or 90 °C) and the normal load applied (20 N). Additionally, the stationary friction coefficient was determined by applying the same constant normal load (20 N) and maintaining a rotational speed of 50 rpm for 10 min. All the tribological tests were conducted, at least, in quadruplicate. Subsequently, the wear scars on the steel plates were examined in triplicate using a BX51 microscope (Make: Olympus, Japan), and the average wear diameters were determined by analyzing the collected images.

Calorimetry tests were accomplished in a Q-50 DSC apparatus (Make: TA Instruments, USA) from 50 to 300 °C. The oxidation onset temperature (OOT) was calculated following the ASTM Standard E2009,^44^ which gives an idea of the initial temperature at which the oxidative degradation of the samples starts to take place.

### 2.5 Statistical Analysis

An analysis of variance (ANOVA) was performed using, at least, three replicates of each measure independently. The means comparison test was also carried out to detect significant differences (*p* < 0.05).

## 3 Results and Discussion

### 3.1 Morphology of Electrospun PKL/CA Nanofiber Mats

**Figure 1:**
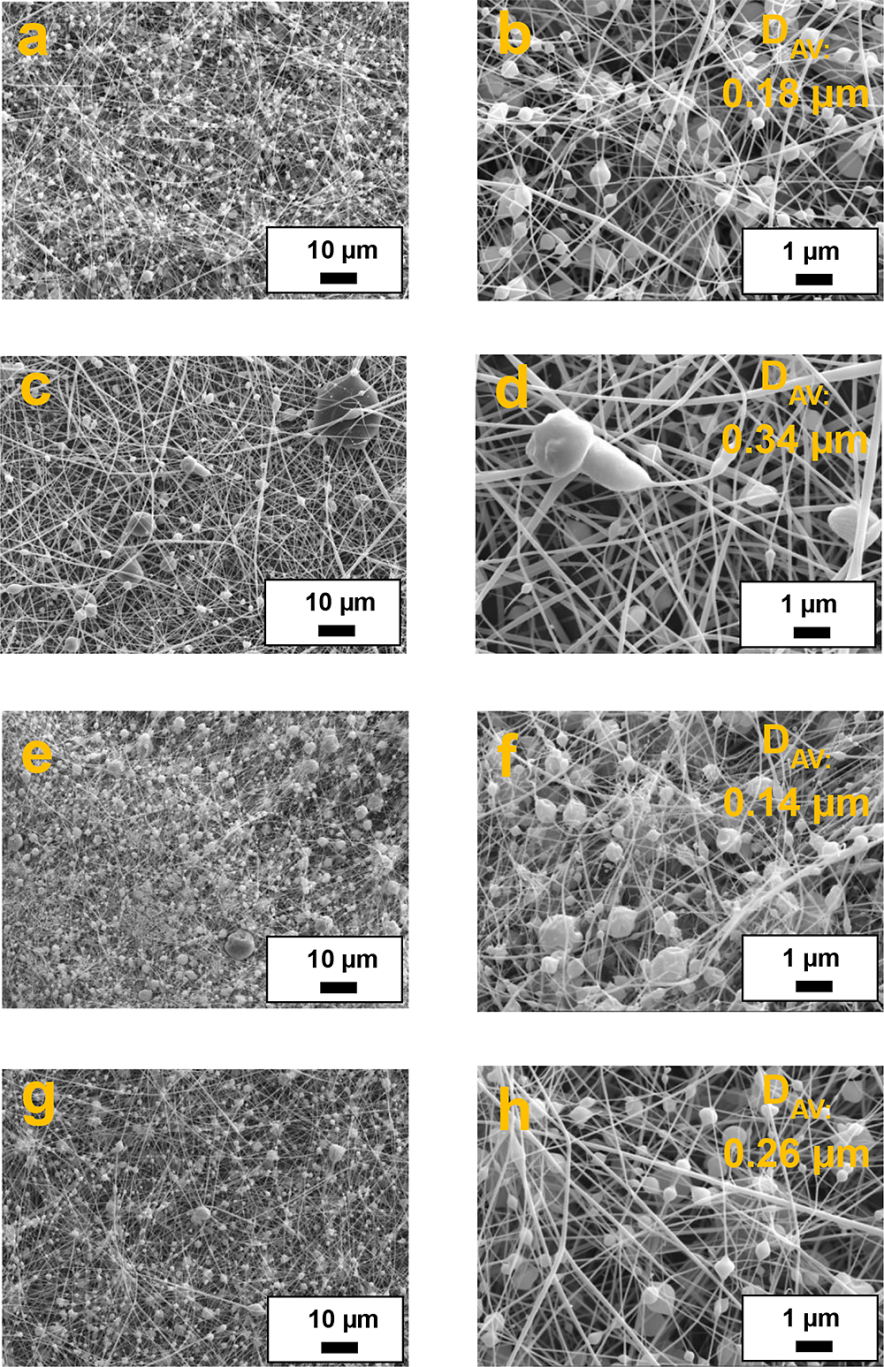
SEM images of electrospun PKL/CA nanostructures as a function of poplar lignin genotype and precipitation pH: (a, b) PO-2.5, (c, d) PO-5, (e, f) Ba-2.5, (g, h) Ba-5.

Figure 1 displays the SEM images of the nanofibers obtained by electrospinning of PKL/CA solutions differing in poplar lignin genotype and precipitation pH. As previously reported,^40^ lignin-only solutions do not generally produce homogeneous nanofiber mats due to the lack of sufficient molecular entanglement as a result of its low molecular weight and heterogeneous chemical structure.^45^ To overcome this problem, the use of a dopant polymer, such as cellulose acetate, that helps to stabilize the jet formation is often required.^40,46^ In this way, the number of isolated particles and beads is significantly reduced. Moreover, the enhanced hydrogen bonding between lignin hydroxyl groups and CA acetyl groups favors fiber formation.^47^ In any case, as shown in Figure 1, beads-on-string (BOAS) structures are generally obtained from PKL/CA solutions, in which beads of sizes below 1 μm are predominantly found on thin filaments. Figure 1 also includes the average diameters of the nanofibers estimated from the analysis of SEM images. The higher the pH of lignin precipitation, the higher the fiber mean diameter, which can be associated with a more linear structure of lignins, as can be inferred from the contents of β-O-4′ and vinyl-ether linkages (see Table 1). As previously reported,^38^ the higher content in these aryl-ether linkages is at the expense of more branched carbon–carbon substructures such as resinol, phenylcoumaran and stilbene. The number of beads appearing in BOAS structures also decreased as the pH of lignin precipitation increased, again favored by a higher content in β-O-4′ and vinyl-ether substructures. On the contrary, electrospun beaded fibers based on PO-2.5 and PO-5 (Figure 1a,b and c,d, respectively) display only slightly thicker fibers than those obtained with their Ballotino genotype counterparts, i.e., Ba-2.5 and Ba-5 samples (Figure 1e,f and g,h respectively). Therefore, poplar lignin genotype does not have a significant impact on the average diameter of electrospun fibers.

### 3.2 Structuring Castor Oil with Electrospun PKL/CA Nanofibers

**Figure 2:**
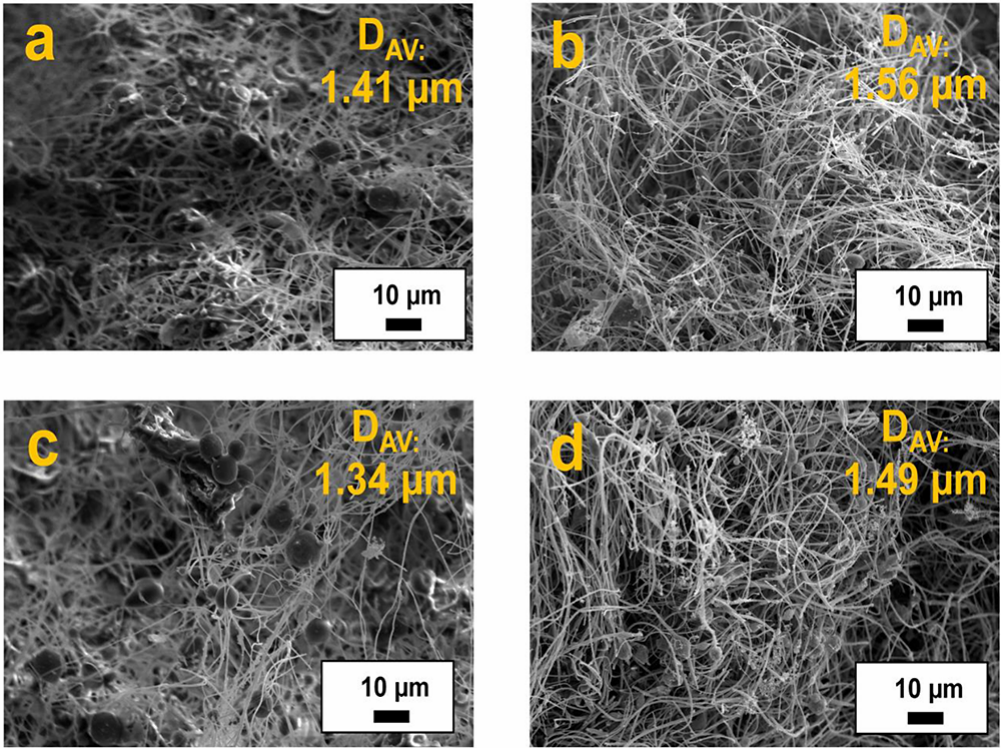
SEM observations of the electrospun PKL/CA nanofibers once dispersed in castor oil as a function of poplar lignin genotype and precipitation pH: (a) O-PO-2.5, (b) O-PO-5 (c), O-Ba-2.5, and (d) O-Ba-5. The codes applied to refer to the oleo-dispersion samples are the lignin codes preceded by O-.

As recently pointed out,^20,40^ nanostructures composed primarily of electrosprayed particles of lignin result in physically unstable dispersions when dispersed in castor oil, while homogeneous electrospun nanofiber mats and BOAS structures demonstrated the ability to form physically stable oleo-dispersions. This stability was achieved through the formation of percolation networks as a result of the increased physical interactions facilitated by the nanofibers’ elevated specific surface area and aspect ratio, which allows the oil to be retained in the porous nanostructure. Similarly, in this work, electrospun PKL/CA nanofiber mats developed from different poplar lignins isolated by precipitation at different pHs were readily dispersed in castor oil, yielding gel-like formulations with a visual appearance that resembles those of conventional lubricating greases or other biobased greases based on NCO-functionalized cellulosic material.^48^Figure 2 shows the SEM morphologies of the resulting gel-like compositions, achieved by dispersing 15 wt % electrospun PKL/CA nanostructures in castor oil, as a function of poplar lignin genotype and precipitation pH. As can be seen, all gel-like dispersions present a rather homogeneous microstructure with uniform nanofiber distribution, where beaded filaments are still easily detectable. Once dispersed in castor oil, nanofibers become more agglomerated and swollen (see the average fiber diameter values inserted in SEM images), but the fiber length are not noticeably altered by the gentle stirring used to disperse the nanostructures in the oil. Considering that castor oil is a moderately polar oil, these findings can be explained by taking into account the hydrophilic nature of poplar Kraft lignin, which facilitates the penetration of castor oil triglycerides into the fibers by a physical mechanism of diffusion, leading to subsequent hydrogen bonding^49^ and swelling. A similar swelling degree has been previously reported in dispersions of electrospun composites of lignocellulosic material derived from spent coffee grounds and postconsumer PET in castor oil, which was mainly attributed to the polar lignocellulosic material in detriment to PET.^50^ Finally, the same slight influence of lignin genotype and pH of precipitation on mean fiber diameter was observed as above-discussed for nanofiber mats directly collected from electrospinning.

**Figure 3:**
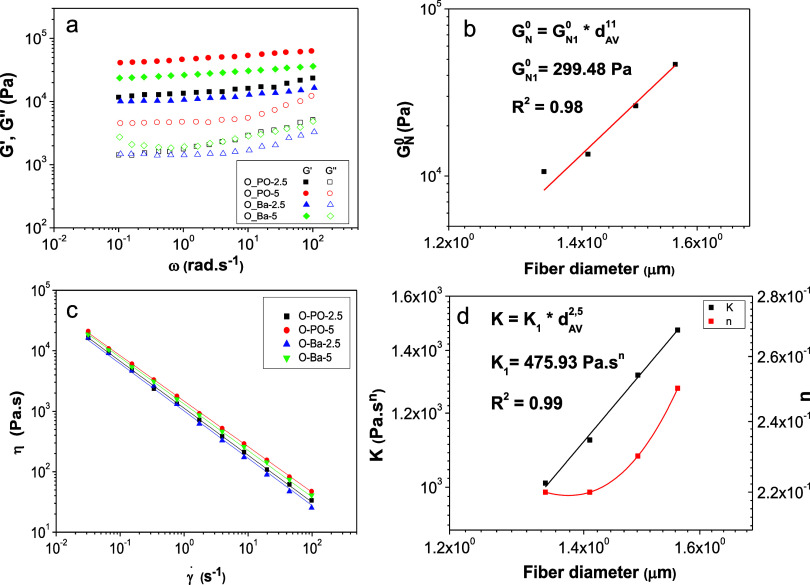
Influence of the poplar lignin genotype and precipitation pH on the rheological properties of electrospun PKL/CA nanofibers’ oleo-dispersions. Evolution of the storage, *G*′, and loss *G*″, moduli with frequency (a); plateau modulus vs average fiber diameter plot (b); viscous flow curves (c); and *K* and *n* vs fiber diameter plots (d).

Figure 3a displays the variation of the SAOS functions, i.e., the storage and loss moduli (*G*′ and *G*″), with frequency for the gel-like formulations obtained by dispersing 15 wt % of electrospun PKL/CA nanofibers in castor oil, as a function of the poplar lignin genotype and precipitation pH. As shown, qualitatively similar mechanical spectra were obtained for all the samples studied. This viscoelastic response is characteristic of gel-like colloidal dispersions, where *G*′ exhibits a slight dependency with frequency and is higher than *G*″. In particular, this viscoelastic behavior was similar to that reported for standard lubricating greases, where *G*′ values typically range from 10^3^ to 10^5^ Pa depending on thickener concentration, being *G*″ values roughly one decade lower.^12,51^ Regarding the genotype and precipitation pH of poplar lignins, the higher viscoelastic functions were obtained by dispersing electrospun PO-5/CA nanostructures, followed by Ba-5/CA, PO-2.5/CA, and Ba-2.5/CA. This is primarily attributed to the differences found in the morphological features of nanofiber mats. In other words, the slight increments found in the average fiber diameter, and the reduction in the number of beads in the BOAS structures, exert a noticeable influence on *G*′ and *G*″ values, with differences of almost one decade between samples O-PO-5 and O-Ba-2.5. On the other hand, the plateau modulus (*G*_N_^0^), which is the characteristic rheological parameter of this type of mechanical spectrum, can be considered a measure of entanglement density and gel strength.^52^ As shown in Figure 3b, *G*_N_^0^ potentially increases with the average fiber diameter in the percolation network, i.e., considering the swollen fibers. This relationship may be described by the simple power-law equation shown in the inset of Figure 3b.

Regarding the viscous flow response, Figure 3c displays the viscosity vs shear rate plots for the gel-like dispersions prepared with electrospun nanostructures as a function of the poplar lignin genotype and precipitation pH. In the shear rate range studied, a shear thinning behavior was always noticed, which can be fairly well described by the classical power-law model:
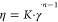
1where *K* and *n* are the consistency and flow indexes, respectively. *K* and *n* values resulting from the fitting to eq 1 are plotted in Figure 3d. As illustrated for *G*_N_^0^, *K* can be correlated with the average fiber diameter of nanofiber mats, similarly following a power-law dependence, whereas *n* also tends to increase with this parameter. Therefore, the morphology of electrospun nanostructures greatly impact the rheological properties of derived oleo-dispersions, which can be tuned by properly selecting the poplar lignin genotype and/or the pH of selective precipitation. Finally, it is worth mentioning that the rheological response of these gel-like dispersions containing 15 wt % electrospun nanofibers is comparable to that of lithium lubricating greases.^51^

### 3.3 Influence of the Vegetable Oil on the Structuring Properties of Electrospun PKL/CA Nanofibers

**Figure 4:**
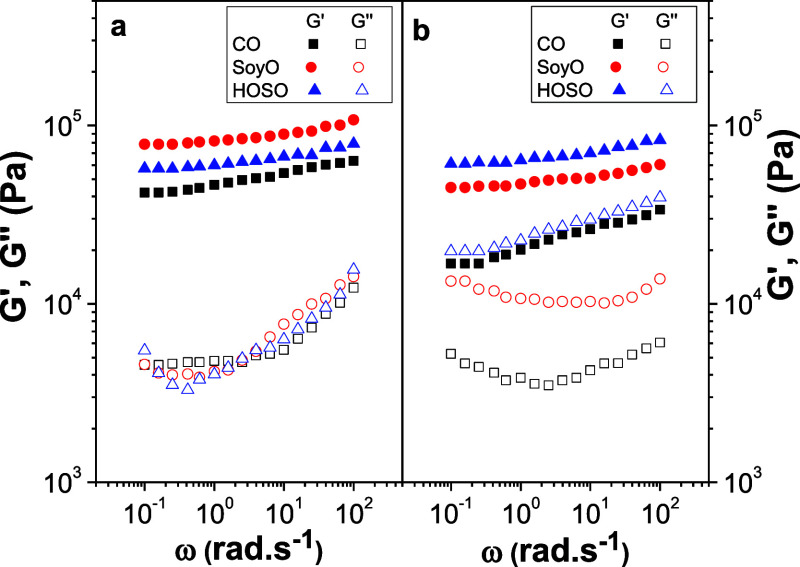
Influence of the vegetable oil on the rheological properties of the electrospun PO-5/CA mats-based gel-like dispersions. Evolution of the storage, *G*′, and loss *G*″, moduli with frequency, at 25 °C (a) and 90 °C (b).

Three different vegetable oils (castor oil (CO), soybean oil (SoyO), and high-oleic sunflower oil (HOSO)) were used to disperse a selected electrospun nanofiber mat (PO-5/CA). As can be found elsewhere,^39^ the main difference between SoyO and HOSO lies in the type of fatty acids prevailing in their compositional profile, which are predominantly polyunsaturated for SoyO and monounsaturated for HOSO, whereas the main difference between CO and these two other vegetable oils is the presence of a hydroxyl group in the predominant ricinoleic acid. As well-known, these hydroxyl groups confer special properties to castor oil such as high polarity and high viscosity, as well as providing a reactive group, which is desired for instance to produce chemical oleogels.^48^ In this case, dispersions of PO-5/CA in both SoyO and HOSO were also able to generate physically stable gel-like formulations with very similar rheological responses, at 25 °C, to that previously discussed for castor oil (see Figure 4a), with values of *G*′ only slightly affected by the vegetable oil. The values of *G*′ decrease as the viscosity of the vegetable oil increases at 25 °C (see viscosity of these vegetable oils elsewhere^39^). A similar effect was reported for standard lithium lubricating greases formulated with paraffinic lubricating oils differing in kinematic viscosity^51^ and oleogels prepared with sorbitan and glyceryl monostearates and several vegetable oils.^53^ However, the values of *G*″ are almost identical.

Bearing in mind the potential application as semisolid lubricants, which generally need to work and maintain their functionality at high temperatures, the viscoelastic properties of gel-like PO-5/CA dispersions in the three vegetable oils were also evaluated at 90 °C (Figure 4b). As can be observed, SAOS functions are greatly affected by temperature for gel-like dispersions based on CO and SoyO, significantly decreasing *G*′ by increasing temperature and shifting the minimum in *G*″ at higher frequencies. However, for those based on HOSO, there is even an increase in the viscoelastic functions which is mainly a consequence of a partial oil release (oil bleeding) observed at that temperature. To a lesser extent, oil release is also responsible for the slightly increased *G*″ values at 90 °C for PO-5/CA dispersion in SoyO. Instead, oil release was not observed for CO, which must be attributed to the higher polarity and, therefore, higher affinity of this vegetable oil for biopolymers such as PKL and CA. In addition, the stronger influence of temperature on the linear viscoelastic functions of CO-based dispersions may also be related to the higher viscosity-temperature dependency, i.e., lower viscosity index, of castor oil.^39^

### 3.4 Antioxidant Properties of Electrospun PKL/CA Nanofibers

The relatively poor oxidation resistance of vegetable oils has hindered the progression toward environmentally friendly lubricating greases.^10,54^ To mitigate this adverse impact, previous approaches have included chemical modifications, the incorporation of antioxidants, or blending with alternative oils like polyalphaolefins (PAO).^44,54,55^ The antioxidant properties of lignin, mainly related to their phenolic content, have been widely demonstrated,^56^ which has led to lignin being tested as a natural antioxidant additive in vegetable oil-based biolubricant formulations.^10^ This fact, combined with the oil structuring capacity previously discussed, makes lignin a potential multifunctional ingredient for semisolid lubricants. In this study, the ASTM standard (E2009) was used to assess the oxidation onset temperature (OOT) of gel-like dispersions based on electrospun PKL/CA nanostructures. The OOT values obtained from calorimetry tests, compared with those found in the literature for lithium and calcium lubricating greases and a cellulose nanofiber-based biolubricant, are shown in Table 2. The OOT values measured for the neat vegetable oils are also included in this Table for reference. The antioxidant properties of lignin can be easily inferred from the higher OOT values generally obtained for the electrospun PKL/CA-structured formulations in comparison with the corresponding neat vegetable oils. The antioxidant action of PKL on castor oil is comparable, or even superior, to that reported for well-known additives such as propyl gallate or 4,4′-methylenebis(2,6-di*tert*-butylphenol).^44^

**Table 2:** Oxidation Onset Temperature (OOT) Values for Gel-Like Dispersions of Electrospun PKL/CA Nanostructures in Different Vegetable Oils (CO, SoyO, and HOSO), Compared with the Respective Neat Vegetable Oils, Commercial Lubricating Greases, and a Model Cellulose Nanofiber-Based Lubricant

samples	OOT (°C)	base oil	relative OOT increment (%)^c^
O-PO-2.5	251	CO	17.3
O-PO-5	256	CO	19.6
O-Ba-2.5	251	CO	17.3
O-Ba-5	258	CO	20.6
O-PO-5/SoyO	187	SoyO	7.5
O-PO-5/HOSO	210	HOSO	5.5
neat CO	214	CO	
neat SoyO	174	SoyO	
neat HOSO	199	HOSO	
lithium soap lubricating grease^a^	207	mineral	
calcium soap lubricating grease^a^	236	mineral	
cellulose nanofiber-based lubricant^b^	194–214	CO	

a Data taken from ref (58).

b Data taken from ref (59).

c Respecting to the neat oils.

The type of poplar Kraft lignin genotype and precipitation pH do not exert a significant influence on the OOT (Table 2), which is somehow an expected result since the electrospun nanofiber concentration is always the same and the composition for all lignin samples is rather similar (see Table 1). The slightly higher OOT values provided by lignins precipitated at pH 5 correlate with higher total phenol contents. On the other hand, the OOT values obtained for the samples based on CO are much higher compared to those based on SoyO and HOSO oils, in agreement with the OOT values obtained for the neat oils, being the most polyunsaturated oil (SoyO) that showing the higher tendency to oxidation. In addition, the relative increment of OOT values induced by the antioxidant action of electrospun lignin nanofibers is much higher in CO (see Table 2).

Finally, as can be deduced from the values collected in Table 2, the oleo-dispersions formulated by dispersing electrospun PKL/CA nanostructures in vegetable oils generally exhibit an oxidation resistance comparable to or even better than lithium and calcium lubricating greases formulated with mineral oils, especially those based on castor oil. Moreover, the antioxidant properties of lignin are again highlighted when comparing these systems with other semisolid biolubricants thickened with cellulose nanofibers (see Table 2), or with other dispersions of modified biopolymers in castor oil, such as *N*-acylated chitosan,^57^ from which no antioxidation properties are expected. Therefore, electrospun PKL/CA nanostructures, besides being validated as suitable oil structuring agents, can be considered a potential multifunctional ingredient with effective antioxidant properties in eco-friendly semisolid lubricant formulations.

### 3.5 Tribological Performance of Gel-Like Dispersions of Electrospun PKL/CA Nanostructures in Vegetable Oils

**Figure 5:**
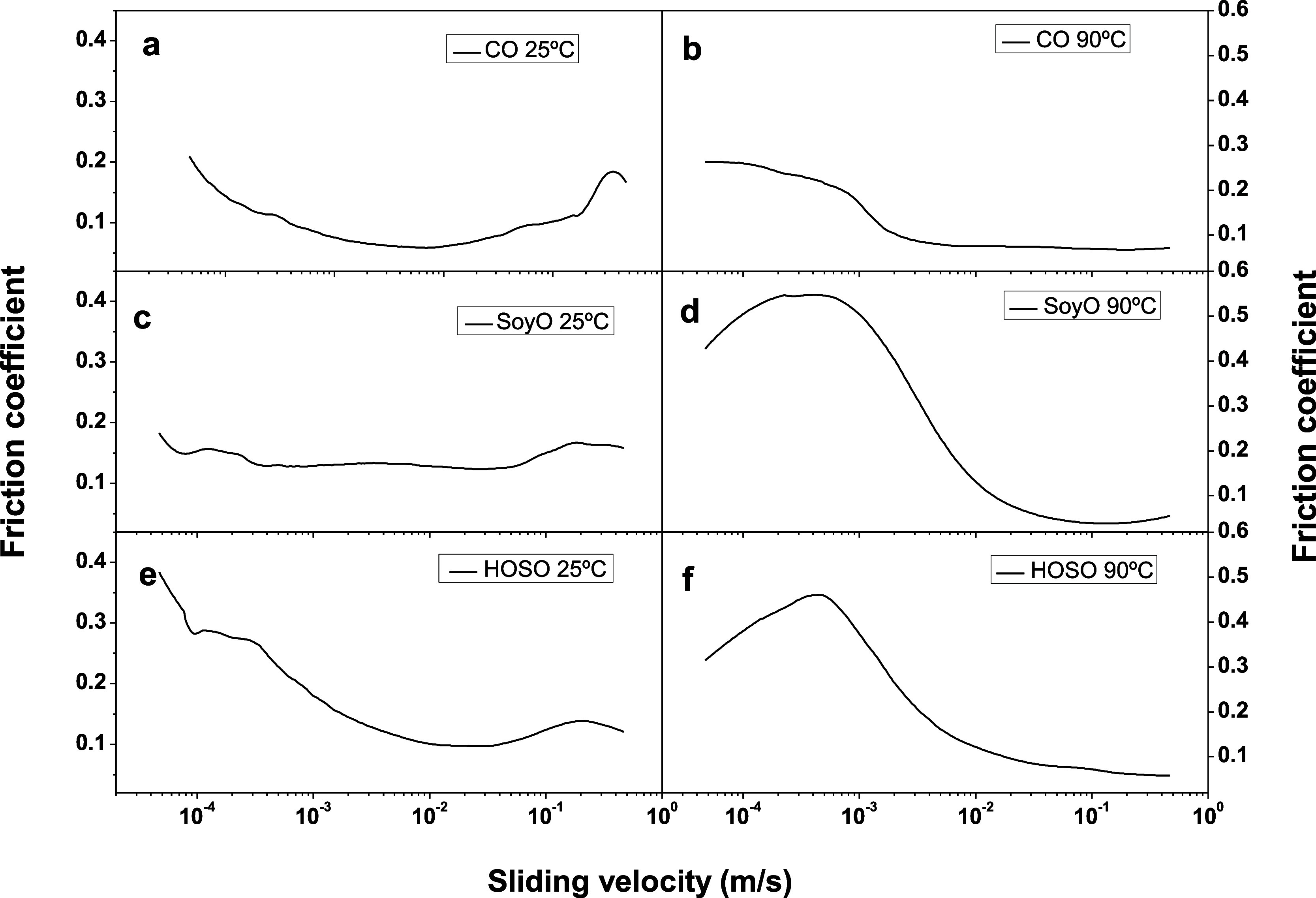
Friction coefficient vs sliding velocity plots (normal force: 20 N; temperature: 25 and 90 °C, respectively) when using the electrospun PO-5/CA mat-based gel-like dispersions in different vegetable oils (CO, SoyO, and HOSO) as lubricants: CO at 25 °C (a) and 90 °C (b), SOyO at 25 °C (c) and 90 °C (d) and HOSO at 25 °C (e) and 90 °C (f).

Considering the potential applicability of the gel-like dispersions of electrospun PKL/CA nanostructures in vegetable oils as semisolid lubricants, the lubrication performance was assessed in a tribological steel–steel ball-on-plates contact. Figure 5 shows the friction coefficient versus sliding velocity plots curves obtained at 25 and 90 °C, under 20 N normal force, using dispersions of PO-5/CA nanofibers in different vegetable oils (CO, SoyO, and HOSO) as lubricants. As can be observed, at 25 °C, a progression from the mixed to the hydrodynamic lubrication regimes can be observed, whereas, at 90 °C, only the decreasing part of the friction coefficient curve is noticed, which encompasses the transition from the boundary to the mixed lubrication regimes. This shift in the appearance of the lubrication regimes with the sliding velocity at high temperatures has been previously reported for greases thickened with several biopolymers^60^ and described on the basis of a reduction in lubricant viscosity and/or viscoelastic properties. Particularly high values of the friction coefficient were measured at low sliding velocities and 90 °C for SoyO and HOSO. These results may be explained on the basis of the lower compatibility of PKL/CA nanofiber with SoyO and HOSO, especially at high temperatures, which results in oil separation to a certain extent, as above-discussed. This fact may favor that only the bled oil comes into the tribological contact and hinders the entrance of the nanofibers, which are likely to be accumulated at the inlet zone, especially when testing under pure sliding conditions. On the contrary, at low temperatures, or using CO as base oil, PKL/CA nanofibers remain well dispersed and may penetrate into the contact more easily, increasing thickness of the lubricating film and reducing friction, even at low sliding velocities.

**Figure 6:**
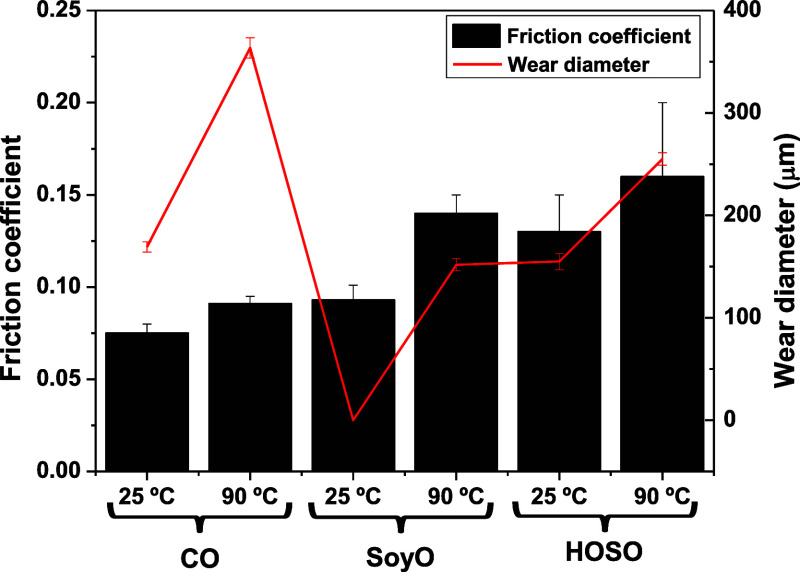
Stationary friction coefficient values and resulting average wear scar diameters obtained by applying a constant sliding velocity (0.023 m/s) and normal load (20 N), at 25 and 90 °C, when using the gel-like dispersions of electrospun PKL/CA nanostructures in different vegetable oils (CO, SoyO, and HOSO) as lubricants.

Moreover, friction coefficient values over time were recorded, at 25 and 90 °C, by applying constant normal load and sliding velocity (20 N and 0.023 m/s), in the mixed lubrication regime. The average stationary values (obtained after 2.5–3 min), as well as the average diameters of the wear scars generated on the plates upon completion of the friction test, are displayed in Figure 6. The images of the wear scars obtained by optical microscopy are included in the Supporting Information (Figure S1). As can be seen, all the samples display reasonably low values of the friction coefficient, which increases with temperature. Moreover, the friction coefficient varies as a function of the type of vegetable oil employed, in this order CO < SoyO < HOSO, because of the higher viscosity and polar character of castor oil. In addition, wear scar diameters obtained on the steel plates were generally comparable to those measured when using standard lubricating greases^60^ or chemical oleogels structured with chemically modified lignocellulosic materials^61,62^ under similar conditions. Interestingly, negligible wear scars were obtained at 25 °C when using SoyO to disperse the nanofibers. It must be noted that PKL/CA nanofibers dispersed in soybean oil show higher values of *G*′ at 25 °C (see Figure 4a), and it may be hypothesized to form a sufficiently robust lubricating film that effectively separates the contacting surfaces, resulting in negligible wear.

## 4 Concluding Remarks

This work explores the feasibility of using poplar lignins (PKL) from different genotypes and isolated at two pHs (5 and 2.5) to produce electrospun nanofibers, in combination with cellulose acetate (CA), with oil structuring ability. This approach represents a new valorization pathway for these waste materials.

Electrospinning PKL/CA solutions produce beaded nanofibers, in which beads of sizes below 1 μm are predominantly found on thin filaments. The number of beads decreases as the pH of lignin precipitation increases, while the average nanofiber diameter increases. Poplar lignin genotype does not exert a significant influence on nanofiber diameter.

Dispersions of 15 wt % electrospun PKL/CA nanofibers in vegetable oils yield physically stable gel-like percolation networks with viscoelastic and shear-thinning characteristics. Once dispersed in castor oil, beaded nanofibers become more agglomerated and swollen.

The morphology of the electrospun nanostructures greatly impact the rheological properties of derived gel-like dispersions, which can be tuned by properly selecting the poplar lignin genotype and/or the precipitation pH. The values of the SAOS functions and shear viscosity increase with the pH of lignin selective acid precipitation or by selecting the *Populus alba* L. genotype to produce the electrospun nanofibers. The plateau modulus and the consistency index correlate potentially with the average fiber diameter of the nanofibers. Besides, the values of *G*′ decrease as the viscosity of the vegetable oil increases. The friction coefficient in a tribological lubricated contact varies as a function the type of vegetable oil employed in this order CO < SoyO < HOSO.

In addition, the antioxidant properties of lignin nanofibers are demonstrated through the much higher OOT values obtained as compared with the corresponding neat vegetable oils. This antioxidant activity is higher in CO rather than in SoyO and HOSO.

In general, the rheological and tribological response of PKL/CA nanofiber dispersions in vegetable oils, including wear prevention, is comparable to those shown by conventional lubricating greases. This allows them to be proposed as environmentally friendly alternatives to the latter, and electrospun PKL/CA nanofibers as a multifunctional ingredient in this kind of formulations.

Finally, the use of electrospun lignin nanofibers as oil structurants still presents some challenges and limitations, primarily related to the electrospinning process, which should be addressed in future research. The utilization of deleterious solvents, such as DMF, to dissolve lignin indeed represents a significant drawback, and there is considerable scope for advancement in the pursuit of suitable environmentally friendly solvents for electrospinning. In this regard, future research may explore alternatives such as ionic liquids and natural deep eutectic solvents (NADES). These alternatives could provide more sustainable and effective solutions to overcome the current limitations, thereby promoting the development of greener and more efficient lignin nanofibers for industrial applications.

## Supporting Information Available

Optical microscopy images of wear scars (PDF)
