# Supplementary material for: Valorization of Kraft Lignins from Different Poplar Genotypes as Vegetable Oil Structuring Agents via Electrospinning for Biolubricant Applications
Source: ACS Sustain Chem Eng. 2024 Jul 29;12(32):12260–9. doi: 10.1021/acssuschemeng.4c05013 (PMC11323950; doi:10.1021/acssuschemeng.4c05013)
Supplement: Supplementary file 1 — sc4c05013_si_001.pdf [file sc4c05013_si_001.pdf]

Supporting Information

Valorization of Kraft lignins from different poplar genotypes as vegetable oil structuring agents via electrospinning for biolubricant applications

José. F. Rubio-Valle, Concepción Valencia, M. Carmen Sánchez-Carrillo, José. E. Martín-Alfonso, José. M. Franco ✉

Pro2TecS – Chemical Product and Process Technology Research Center. Department of Chemical Engineering and Materials Science. Universidad de Huelva. ETSI. Campus de "El Carmen". 21071 Huelva. Spain.

✉ Corresponding author:

Prof. José M. Franco, Dept. Chemical Engineering and Materials Science, ETSI, Campus de "El Carmen". Universidad de Huelva, 21071 Huelva, Spain.
Phone: +34 959 219995; e-mail: franco@uhu.es

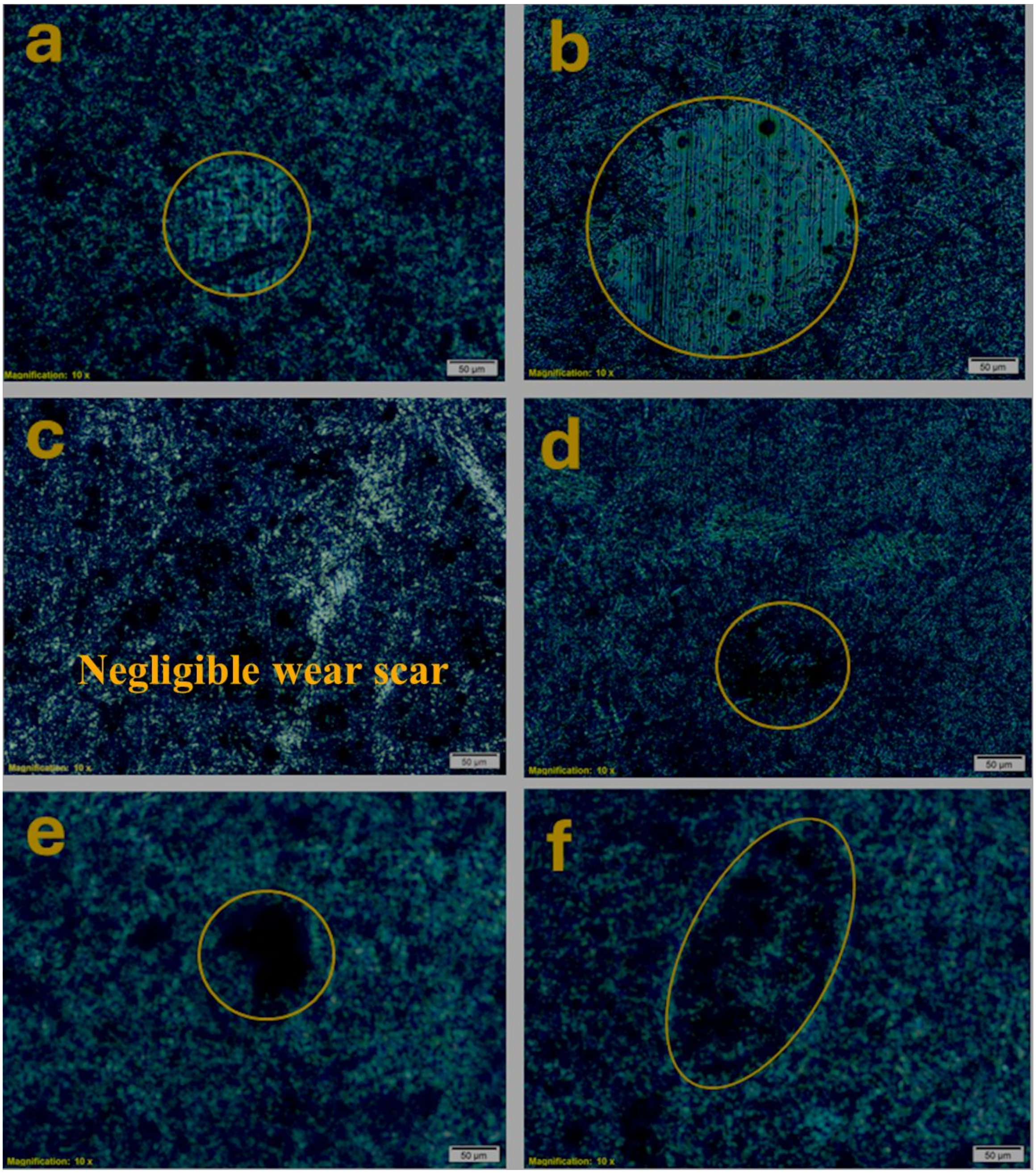

Figure S1. Optical microscopy images of wear scars obtained when using the gel-like dispersions of electrospun PKL/CA nanostructures in different vegetable oils (CO, SoyO and HOSO) as lubricants: CO at 25°C (a) and 90°C (b), SOyO at 25°C (c) and 90°C (d) and HOSO at 25°C (e) and 90°C (f).
